# Apolipoprotein A‐IV acts as an endogenous anti‐inflammatory protein and is reduced in treatment‐naïve allergic patients and allergen‐challenged mice

**DOI:** 10.1111/all.14022

**Published:** 2019-09-10

**Authors:** David Roula, Anna Theiler, Petra Luschnig, Gunter J. Sturm, Peter V. Tomazic, Gunther Marsche, Akos Heinemann, Eva M. Sturm

**Affiliations:** ^1^ Division of Pharmacology, Otto‐Loewi Research Center for Vascular Biology, Immunology and Inflammation Medical University of Graz Graz Austria; ^2^ Department of Dermatology and Venerology Medical University of Graz Graz Austria; ^3^ Allergy Outpatient Clinic Reumannplatz Vienna Austria; ^4^ ENT‐University Hospital Medical University of Graz Graz Austria

**Keywords:** allergic inflammation, apolipoprotein A‐IV, chronic rhinosinusitis, eosinophils, house dust mite‐induced asthma model, PI3‐kinase, Rev‐Erb‐α

## Abstract

**Background:**

Recent studies pointed to a crucial role for apolipoproteins in the pathogenesis of inflammatory diseases. However, the role of apolipoprotein‐IV (ApoA‐IV) in allergic inflammation has not been addressed thoroughly thus far.

**Objective:**

Here, we explored the anti‐inflammatory effects and underlying signaling pathways of ApoA‐IV on eosinophil effector function in vitro and in vivo.

**Methods:**

Migratory responsiveness, Ca^2+^‐flux and apoptosis of human peripheral blood eosinophils were assessed in vitro. Allergen‐driven airway inflammation was assessed in a mouse model of acute house dust mite‐induced asthma. ApoA‐IV serum levels were determined by ELISA.

**Results:**

Recombinant ApoA‐IV potently inhibited eosinophil responsiveness in vitro as measured by Ca^2+^‐flux, shape change, integrin (CD11b) expression, and chemotaxis. The underlying molecular mechanism involved the activation of Rev‐ErbA‐α and induced a PI3K/PDK1/PKA‐dependent signaling cascade. Systemic application of ApoA‐IV prevented airway hyperresponsiveness (AHR) and airway eosinophilia in mice following allergen challenge. ApoA‐IV levels were decreased in serum from allergic patients compared to healthy controls.

**Conclusion:**

Our data suggest that ApoA‐IV is an endogenous anti‐inflammatory protein that potently suppresses effector cell functions in eosinophils. Thus, exogenously applied ApoA‐IV may represent a novel pharmacological approach for the treatment of allergic inflammation and other eosinophil‐driven disorders.

## Graphical abstract

ApoA‐IV is decreased in serum from untreated allergic patients compared to healthy controls. ApoA‐IV potently inhibits eosinophil function in vitro. Systemic application of ApoA‐IV reduces airway hyperreactivity and airway eosinophilia in a murine model of allergic asthma. ApoA‐IV, apolipoprotein A‐IV; HDM, house dust mite

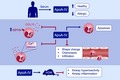

## 1 INTRODUCTION

Eosinophils make up to 5% of circulating human blood leukocytes in healthy individuals and normally survive up to two days in blood. However, this period may be extended by eosinophil‐activating cytokines under inflammatory conditions such as infectious and allergic diseases.^1^ Activated eosinophils are a major source of reactive oxygen species (ROS), cytotoxic proteins, and proinflammatory cytokines,^2^ and thereby modulate the immune microenvironment and promote several immunoregulatory functions. Eosinophils are involved in antigen‐presentation^3^ and T‐cell activation,^4^ interact with and activate other immunocompetent cells such as dendritic cells,^5^ mast cells,^6^ macrophages,^7^ and neutrophils.^8^ Moreover, activated eosinophils signal to and activate resident tissue cells such as epithelial cells,^9^ endothelial cells,^10^ goblet cells,^11^ smooth muscle cells,^12^ fibroblasts,^13^ and neurons,^14^ overall leading to the progression of inflammation, mucus secretion, tissue remodeling, and angiogenesis.^15^ Thus, eosinophils are potent effectors and modulators of various diseases ranging from bronchial asthma^16^ and atopic dermatitis^17^ to eosinophilic esophagitis,^18^ colitis ulcerosa^19^ and hypereosinophilic syndrome.^20^ In asthmatics, levels of eosinophil granule proteins such as eosinophil cationic protein (ECP) or eosinophil peroxidase (EPO) largely correlate with disease severity.^21^ Moreover, patients who receive treatment based on eosinophil counts in sputum have significantly fewer exacerbations than patients treated according to standard therapy.^22^ Of note, eosinophilic inflammation of the upper airways may also occur independent of allergy as observed in chronic rhinosinusitis (CRS) patients.^23^ Similar to allergies, CRS causes not only physical suffering, but also impacts psychological well‐being and daily functioning. Patients with eosinophilic CRS represent a unique subtype and remain largely resistant to medical and surgical interventions. Thus, therapies that specifically target eosinophilic expansion and effector functions are urgently needed.

The apolipoprotein ApoA‐IV is—to some extent—found on chylomicrons and HDL in plasma; however, its lipid‐free form is predominant in circulation,^24^ where it is presumed to play anti‐inflammatory roles. In fact, the expression of human ApoA‐IV in ApoE−/− mice protected them from oxidative stress, decreased the secretion of proinflammatory cytokines after LPS administration and reduced the formation of atherosclerotic lesions.^25^, ^26^ Furthermore, in an experimental model of DSS‐induced colitis, ApoA‐IV inhibited leukocyte and platelet adhesive interactions and suppressed the upregulation of P‐selectin on colonic endothelium.^27^ In humans, ApoA‐IV was found to inhibit histamine release from basophils in vitro,^28^ and, interestingly, ApoA‐IV levels increased in the blood of allergic rhinitis patients following sublingual immunotherapy and were inversely correlated with symptom‐medication scores.^28^

In this study, we set out to explore whether the anti‐inflammatory properties of ApoA‐IV also extend to eosinophil effector function in vitro and in a mouse model of allergen‐induced pulmonary inflammation. Moreover, we assessed ApoA‐IV levels in patients with eosinophil‐driven diseases such as allergy and chronic rhinosinusitis. Our data clearly indicate that ApoA‐IV is a potential resolution factor in eosinophilic inflammation and might have beneficial effects on eosinophil‐driven diseases.

## 2 METHODS

Detailed description of patient cohorts, ethical permits, materials, and procedures is provided in the Methods section in this article's Online Repository (Appendix S1).

### 2.1 Isolation peripheral blood eosinophils

Human peripheral blood eosinophils were isolated from citrated whole blood from allergic or healthy donors. In brief, erythrocytes were removed by dextran sedimentation and polymorphonuclear leukocytes (pellet) were separated from mononuclear cells (buffy coat) by density gradient centrifugation using Histopaque 1077. Eosinophils were separated from neutrophils in the polymorphonuclear leukocyte fraction by negative magnetic selection using the MACS cell separation system (Eosinophil Isolation Kit; Miltenyi Biotec, Bergisch Gladbach, Germany) with a resulting purity of typically ≥ 98%.^29^

### 2.2 Shape change assay

Eosinophil shape change was assessed in polymorphonuclear leukocyte (PMNL) preparations and monitored by flow cytometry as an increase in the forward scatter signal.^30^

### 2.3 CD11b‐upregulation

To assess CD11b upregulation, PMNL samples or citrated whole blood were stained with PE‐Cy5‐anti‐CD16 and PE‐anti‐CD11b Abs and measured by flow cytometry.^31^

### 2.4 Calcium flux

Intracellular Ca^2+^ release from purified human eosinophils was detected by flow cytometry using the Ca^2+^ sensitive dye Fluo 3‐AM.^29^

### 2.5 Chemotaxis

Eosinophil chemotaxis experiments were done with purified eosinophils, whereas neutrophil chemotaxis was performed in separate experiments with PMNL preparations. Chemotaxis assays were performed in a 48‐well micro chemotaxis chamber using PVP‐free polycarbonate filters with a pore size of 5 µm. Migrated cells were enumerated by flow cytometry.^32^ Therefore, eosinophils and neutrophils were gated by their forward and side scatter properties and by autofluorescence.

### 2.6 Cholesterol‐rich microdomain (lipid raft) assessment

Lipid raft abundance was quantified by flow cytometry in purified eosinophils stained with FITC‐cholera toxin B.^33^

### 2.7 CCR3 staining

CCR3 expression was evaluated by flow cytometry in PE‐anti‐CD193 (CCR3) stained purified eosinophils.

### 2.8 Apoptosis assay

Purified eosinophils were stained with FITC‐annexin‐V/PI and analyzed by flow cytometry.

### 2.9 House dust mite‐induced allergic lung inflammation

The HDM model was performed as described by Plantinga et al^34^ In brief, Balb/c mice were sensitized i.n. with 1 μg HDM extract on day 1 and were challenged intranasally with 10 μg HDM per day from day 7 to day 11. On day 15, lung function testing was performed or BAL fluid, bone marrow, and spleens were taken. Leukocytes were analyzed by flow cytometry.

### 2.10 Statistical analysis

Data are shown as mean + or ± SEM for n observations, where n denotes independent experiments with cells from different donors. Comparisons of groups were performed as appropriate using Student's t test or Mann‐Whitney test, 1‐way ANOVA followed by Dunnett's or Tukey's post hoc test or 2‐way ANOVA for repeated measurements followed by Bonferroni's post hoc test to determine the levels of significance for each group. Probability values of *P* < .05 were considered as statistically significant.

## 3 RESULTS

### 3.1 Preliminary experiments

To define the working dose of ApoA‐IV as well as ApoA‐I and HDL for following assays, preparatory chemotaxis experiments were performed. As shown in Figure S1, ApoA‐IV (1‐10 µg/mL), ApoA‐I (1‐30 µg/mL), and HDL (10‐100 µg/mL) concentration‐dependently impaired eosinophil migration toward CCL11 (3 nmol/L). Based on these results, 1‐3 µg/mL ApoA‐IV, 10 µg/mL ApoA‐I, and 100 µg/mL HDL were used in subsequent experiments.

### 3.2 ApoA‐IV impairs eosinophil responsiveness

Since the effects of ApoA‐IV on eosinophils have not been deciphered so far, we first explored the anti‐inflammatory capacity of ApoA‐IV in assays of eosinophil shape change, integrin upregulation, and intracellular Ca^2+^ mobilization. When encountering a chemotactic factor, such as CCL11, eosinophils immediately prepare for diapedesis through the endothelium by rearranging their cytoskeleton. Such morphological changes can be detected by flow cytometry as increases in the forward scatter properties of the cells. We studied the effects of ApoA‐IV on eosinophil shape change in PMNL samples from healthy nonallergic donors. We pretreated samples with recombinant ApoA‐IV or vehicle for 30 minutes, followed by stimulation with serial dilutions of CCL11, and shape change was monitored by flow cytometry. Of particular interest, already very low concentrations of ApoA‐IV (1 µg/mL) led to a statistically significant decrease of eosinophil shape change, as the responsiveness to CCL11 was decreased by 50% (Figure S2A). Besides shape change, upregulation of adhesion molecules such as α_m_β_2_ integrins (CD11b/CD18; Mac‐1) is another precondition for eosinophil migration. To measure the impact of ApoA‐IV on integrin mobilization, we pretreated human eosinophils in PMNL fractions with ApoA‐IV (1 µg/mL) or vehicle and stimulated again with CCL11. Coinciding with the effect on shape change, ApoA‐IV clearly reduced the presence of CD11b molecules on the cell surfaces by 30% (Figure S2B). Beside morphological changes and integrin upregulation, CCL11 induces a rapid and transient rise in intracellular Ca^2+^ ions. Similar to shape change and CD11b, we found that ApoA‐IV reduced this CCL11‐induced Ca^2+^ mobilization in a concentration‐dependent fashion. As presented in Figure S2C, Ca^2+^ flux was diminished by 30% and 45% in the presence of 1 and 3 µg/mL of ApoA‐IV, respectively.

### 3.3 ApoA‐IV inhibits eosinophil chemotaxis

**Figure 1:**
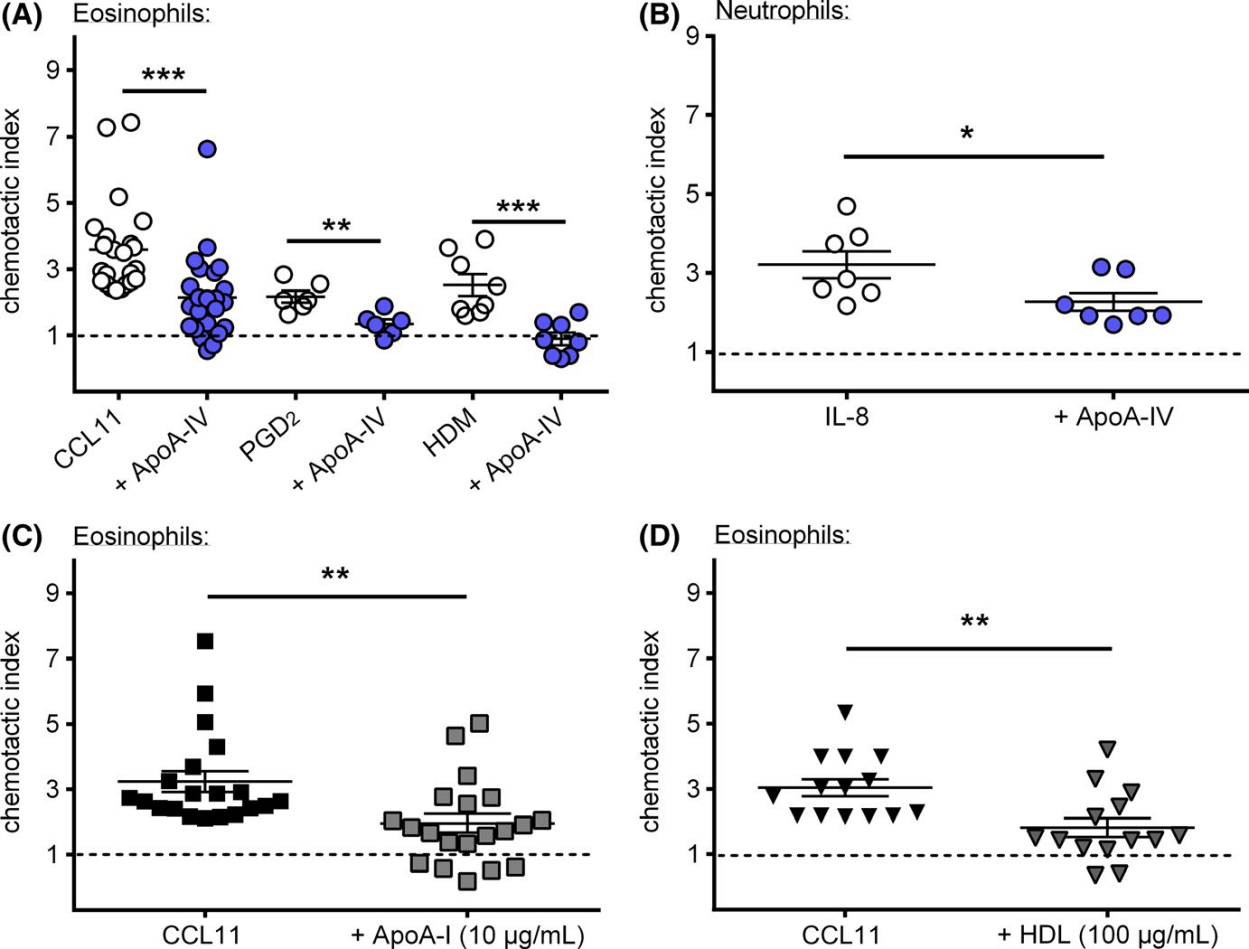
ApoA‐IV potently inhibits eosinophil chemotaxis. (A) Incubation with ApoA‐IV (1 µg/mL) for 30 min reduced eosinophil chemotaxis toward CCL11 (3 nmol/L), PGD_2_ (30 nmol/L) and house dust mite extract (HDM, 100 µg/mL) (n = 7‐23) as well as (B) neutrophil chemotaxis toward IL‐8 (10 nmol/L) (n = 7). Treatment with (C) ApoA‐I (10 µg/mL) (n = 20) and (D) HDL (100 µg/mL) (n = 14) for 30 min similarly decreased eosinophil chemotaxis toward CCL11 (3 nmol/L). Chemotaxis of purified eosinophils and neutrophils in PMNL fractions was performed in duplicates in a 48‐well microchemotaxis chamber. Migrated cells were enumerated by flow cytometry. Data are shown as mean ± SEM; *P < .05, **P < .01, *** P < .01; Student's *t* test

**Figure 2:**
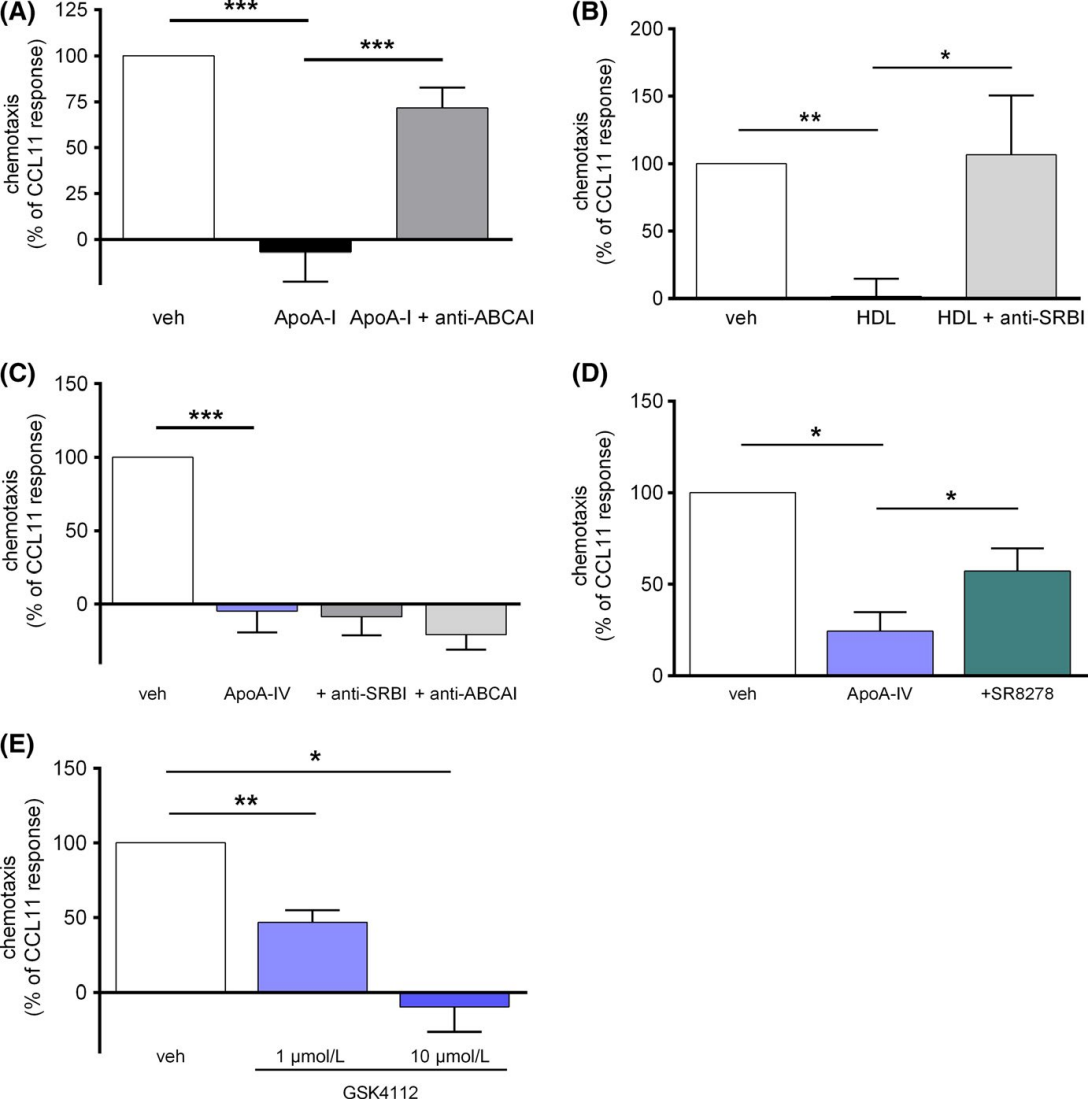
ApoA‐IV acts independently from SRBI and ABCAI but requires the nuclear receptor Rev‐ErbA‐α. (A) Purified eosinophils were pretreated with an anti‐ABCAI blocking antibody for 30 min, incubated with ApoA‐I (10 µg/mL) for 30 min and chemotaxis was stimulated with CCL11 (3 nmol/L) (n = 5). (B) Purified eosinophils were pretreated with an anti‐SRBI blocking antibody for 30 min, incubated with HDL (100 µg/mL) for 30 min and chemotaxis was stimulated with CCL11 (3 nmol/L) (n = 7). (C) Eosinophils were pretreated either with an anti‐ABCAI or anti‐SRBI blocking antibody, incubated with ApoA‐IV (3 µg/mL) for 30 min and chemotaxis was stimulated with CCL11 (3 nmol/L) (n = 4). (D) Eosinophils were pretreated with the Rev‐ErbA‐α antagonist SR8278 (1 µmol/L) for 30 min, incubated with ApoA‐IV (3 µg/mL) for 30 min and chemotaxis was stimulated with CCL11 (3 nmol/L) (n = 5). (E) Eosinophils were incubated with the Rev‐ErbA‐α agonist GSK4112 (1 and 10 µmol/L) for 30 min and chemotaxis was stimulated with CCL11 (3 nmol/L) (n = 5). (A‐E) Purified eosinophils were pretreated as indicated and chemotaxis to CCL11 (3 nmol/L) was determined in duplicates in a 48‐well microchemotaxis chamber. Migrated cells were enumerated by flow cytometry. Data are shown as mean + SEM; *P < .05, ** P < .01, *** P < .001*;* 1‐way ANOVA

Having established that ApoA‐IV affects cellular responsiveness of eosinophils, we next investigated the direct impact of ApoA‐IV—in comparison to ApoA‐I or isolated HDL—on eosinophil migration. Chemotaxis assays were carried out in a modified Boyden chamber using a 48*‐*well microchemotaxis assembly. As displayed in Figure 1A, ApoA‐IV (1 µg/mL) not only inhibited eosinophil chemotaxis toward CCL11 (3 nmol/L), but also toward prostaglandin D_2_ (30 nmol/L) by 56% and 70%, respectively. Similarly, ApoA‐IV even abolished migration toward house dust mite extract (HDM; 100 µg/mL) which has been shown to directly activate and mobilize eosinophils.^35^ For neutrophil chemotaxis toward IL‐8, a reduction of 41% from CI = 3.2 to CI = 2.3 was observed (Figure 2C). Of note, ApoA‐IV (1 µg/mL) (Figure 1A), ApoA‐I (10 µg/mL) (Figure 1C) and HDL (100 µg/mL) (Figure 1D) impaired eosinophil chemotaxis toward CCL11 (3 nmol/L) to a similar extent.

### 3.4 ApoA‐IV acts NR1D1‐dependently and signals via PI3K/PDK1 and PKA

It is assumed that ApoA‐I attenuates neutrophil function via the ATP‐binding cassette transporter AI (ABCAI), whereas anti‐inflammatory effects of HDL are mediated via scavenger receptor BI (SRBI).^36^ Thus, we next scrutinized whether ApoA‐IV also signals via ABCAI or SRBI binding. As shown in Figure 2, ABCAI blocking averted the effect of ApoA‐I (Figure 2A) and the SRBI antibody impeded the HDL‐mediated decrease of eosinophil chemotaxis (Figure 2B). Notably, neither ABCAI nor SRBI blocking could prevent the inhibitory effect of ApoA‐IV (Figure 2C). Thus, ApoA‐IV appears to inhibit eosinophil function through a mechanism different from ApoA‐I and HDL. Recently, the nuclear receptor NR1D1 (Rev‐ErbA‐α) has been identified as a putative ApoA‐IV‐binding protein in hepatocytes.^37^, ^38^ Of note, the Rev‐ErbA‐α antagonist SR8278 (1 µM) partially reversed the ApoA‐IV‐induced inhibition of eosinophil chemotaxis (Figure 2D) and the selective Rev‐ErbA‐α agonist GSK4112 (SR6452) mimicked the impeding effect of ApoA‐IV (Figure 2E).

**Figure 3:**
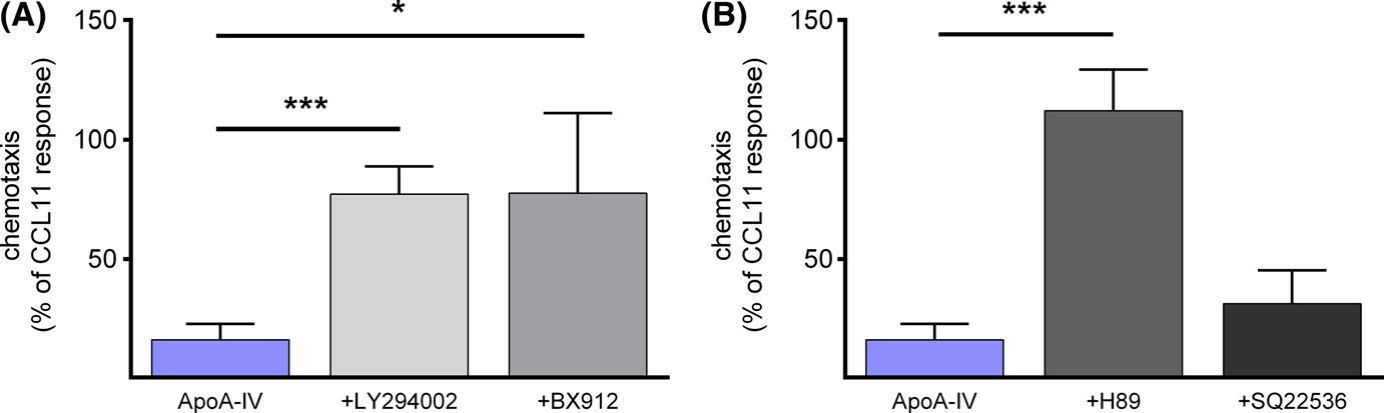
ApoA‐IV involves PI3K/PDK1/PKA activity. (A) Purified eosinophils were pretreated with the PI3K inhibitor LY294002 (20 µmol/L) (n = 6) or the PDK1 inhibitor BX912 (300 nmol/L) (n = 4) for 30 min, incubated with ApoA‐IV (3 µg/mL) for 30 min and chemotaxis was induced by CCL11 (3 nmol/L). (B) Purified eosinophils were pretreated with the PKA inhibitor H89 (1 µmol/L) or the adenylyl cyclase inhibitor SQ22536 (10 µmol/L) for 30 min, incubated with ApoA‐IV (3 µg/mL) for 30 min and chemotaxis was induced by CCL11 (3 nmol/L) (n = 5‐6). (A‐B) Purified eosinophils were pretreated as indicated and chemotaxis was induced by CCL11 (3 nmol/L) and performed in duplicates in a 48‐well microchemotaxis chamber. Migrated cells were enumerated by flow cytometry. Data are shown as mean + SEM; * P < .05, *** P < .001; 1‐way ANOVA

To further elucidate the downstream components of the ApoA‐IV pathway in eosinophils, cells were incubated with protein kinase inhibitors. As illustrated in Figure 3A, blocking PI3K and PDK1 prevented the ApoA‐IV induced inhibition of eosinophil chemotaxis. ApoA‐IV alone reduced eosinophil chemotaxis to ~ 15% of the CCL11 response, whereas pretreatment with the PI3K inhibitor LY294002 (10 μmol/L) and the PDK1 inhibitor BX912 (300 nmol/L) reverted the CCL11‐induced chemotaxis to 77% of the control response. Moreover, eosinophils that were pretreated with the PKA inhibitor H89 (1 µmol/L) even reached 112% of the CCL11‐induced chemotaxis (Figure 3B). In contrast, the adenylyl cyclase inhibitor SQ22536 (10 µmol/L) showed no significant effect. Hence, the anti‐inflammatory activity of ApoA‐IV seems to require PI3K and PDK1 as well as cAMP‐independent activation of PKA. Consistently, PI3K activation has already been associated with other responses to ApoA‐IV.^39^, ^40^

### 3.5 ApoA‐IV neither disrupts lipid rafts nor affects CCR3 surface expression

Given that cholesterol‐rich membrane (lipid) rafts play an important role in leukocyte activation, we investigated whether ApoA‐IV alters lipid raft abundance in eosinophils. For that purpose, cholera toxin B‐FITC that interacts with the raft component ganglioside GM1, was used to quantify lipid rafts by flow cytometry. However, lipid raft integrity was affected neither by ApoA‐IV (3 µg/mL) nor by ApoA‐I (10 µg/mL) treatment for 60 minutes (Figure S3A).

Since the ApoA‐IV‐related apolipoprotein ApoE was found to modulate the expression of proinflammatory molecules such as the CCL11 receptor CCR3 on activated microglia,^41^ we investigated whether eosinophil CCR3 surface expression is altered in response to ApoA‐IV (3 µg/mL). As depicted in Figure S3B, ApoA‐IV did not reduce CCR3 staining after a 60‐minutes treatment.

### 3.6 ApoA‐IV enhances apoptosis in eosinophils from allergic subjects

As apoptotic cell death plays an important role in the resolution of inflammatory reactions, we next examined the ability of ApoA‐IV to modulate apoptosis in eosinophils. Purified cells were incubated for an 18‐hours period with ApoA‐IV (3 µg/mL) or ApoA‐I (10 µg/mL) and apoptosis was assessed by flow cytometry using annexin‐V/PI staining. Annexin‐V/PI dual‐negative cells were considered live cells, annexin‐V‐positive cells were considered apoptotic cells, whereby annexin‐V‐positive/PI‐negative cells were considered early apoptotic and annexin‐V/PI dual‐positive cells were considered late apoptotic cells. PI‐positive cells were considered necrotic cells. Interestingly, ApoA‐IV accelerated eosinophil apoptosis only in allergic donors (Figure S4). After 18 hours of incubation, the percentage of live cells (annexin‐V/PI dual‐negative) decreased from 51.1% ± 3.9 (vehicle treatment) to 34.8 ± 4.6% in ApoA‐IV‐treated eosinophils from allergic donors (Figure S4A). Moreover, ApoA‐IV specifically increased the percentage of apoptotic (PI‐negative and positive) cells from 43 ± 5.2% (vehicle treatment) to 60 ± 2.6% in allergic donors, while ApoA‐I was less effective (increase of apoptotic cells to 48 ± 5.6%) (Figure S4B). No significant differences were observed for early apoptotic (Figure S4C), late apoptotic (Figure S4D), and necrotic cells (Figure S4E).

### 3.7 Systemic application of ApoA‐IV alleviates allergen‐induced airway eosinophilia and airway hyperresponsiveness (AHR) in mice

Having confirmed the anti‐inflammatory properties of ApoA‐IV in vitro, we hypothesized that ApoA‐IV might be effective at inhibiting airway inflammation and hyperresponsiveness in a murine model of house dust mite extract (HDM)‐induced asthma. In brief, eight‐week‐old female Balb/c mice were intranasally immunized to HDM or treated with vehicle (PBS) on day 1 and challenged intranasally with HDM or vehicle from day 7 to day 11. All analyses were performed on day 15.

**Figure 4:**
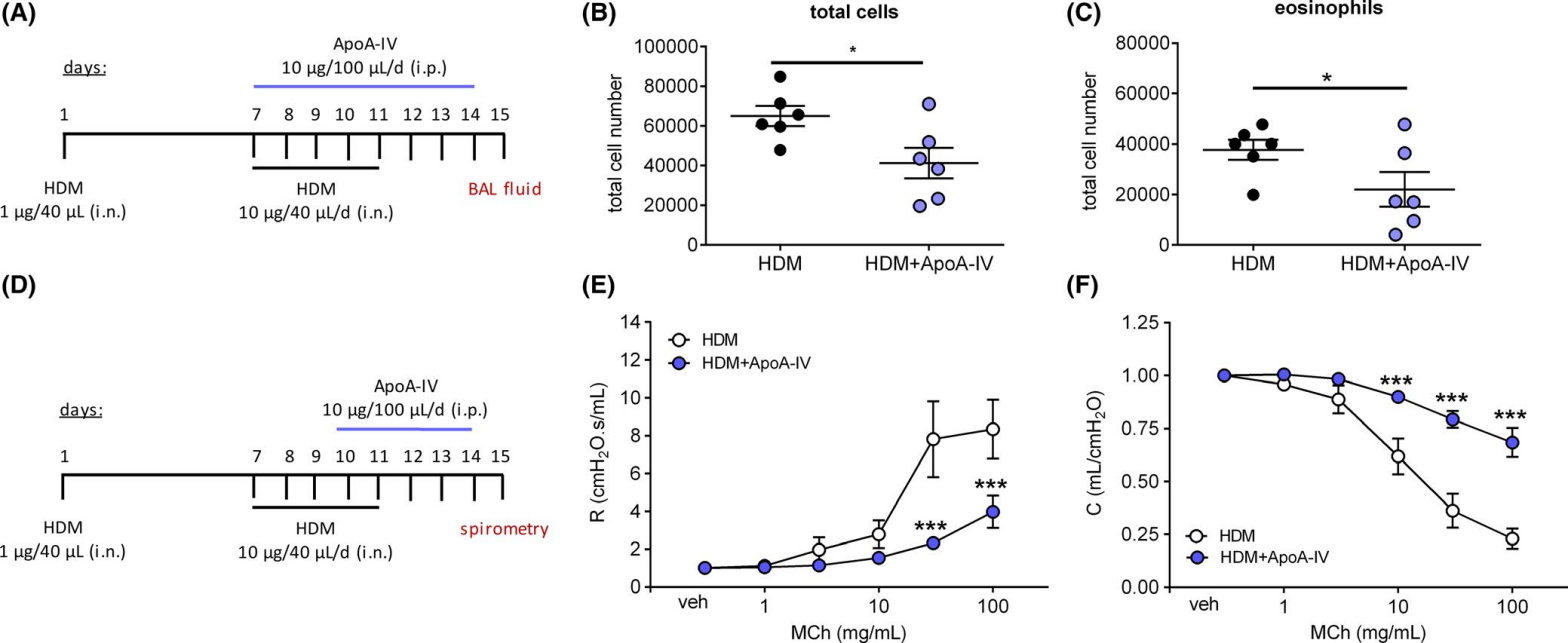
ApoA‐IV reduces HDM‐induced eosinophilia in BAL fluid and improves lung function in HDM‐sensitized mice. (A) On day 1, eight‐week‐old female Balb/c mice were sensitized intranasally with 1 µg HDM and challenged by intranasal application of 10 µg HDM per day from day 7 to 11. From day 7 to 14 mice were daily injected with 10 µg ApoA‐IV or vehicle. On day 15 BAL fluid was taken from six mice per group and cellular contents were analyzed by flow cytometry. For (B) total cell count and for (C) eosinophils in the BAL fluid are shown. (D) On day 1, eight‐week‐old female Balb/c mice were sensitized intranasally with 1 µg HDM and challenged by intranasal application of 10 µg HDM per day from day 7 to 11. From day 10 to 14 mice were daily injected with 10 µg ApoA‐IV or vehicle. On day 15 lung function was assessed while applying increasing doses of methacholine (MCH) by a FlexiVent system and (E) airway resistance and (F) compliance were measured. (B, C) Data are shown as mean ± SEM, * P < .05; Student's *t* test; (E, F) data are shown as mean ± SEM, *** *P* < .001; 2‐way ANOVA; n = 5‐6

First, we compared ApoA‐IV serum levels of HDM‐sensitized/challenged mice with the vehicle‐treated control group. As determined by ELISA, ApoA‐IV serum levels of HDM‐treated mice were significantly decreased by 34% compared to the vehicle‐treated group (Figure S5). Since infiltrating immune cells are main driving forces of allergic airway inflammation, we examined these cells in the BAL fluid of ApoA‐IV‐‐treated and untreated asthmatic mice. Therefore, HDM‐exposed mice received a daily i.p. injection of ApoA‐IV (10 µg/100 µL) or vehicle (A. dest.) from day 7 to day 14 (Figure 4A). As depicted in Figure 4B‐C, ApoA‐IV strongly repressed the infiltration of immune cells, mainly eosinophils, into the bronchoalveolar space. Treatment with ApoA‐IV for 8 days protected mice from airway eosinophilia as reflected by a ~42% reduction in eosinophil counts in the BAL fluid of ApoA‐IV‐treated mice compared to vehicle‐treated controls (Figure 4C). ApoA‐IV also tended to reduce the numbers of alveolar macrophages in the BAL fluid; however, this difference did not reach significance (Figure S6A). Similarly, counts of lymphocytes, monocytes, and neutrophils in the BAL fluid remained unchanged (Figure S6B‐D). Of note, ApoA‐IV supplementation also protected from systemic eosinophilia as reflected by a ~60% reduction of eosinophil counts in spleen tissue (Figure S7A) and in bone marrow (Figure S7B).

Allergic airway inflammation causes various symptoms of asthma such as airflow obstruction, which is usually associated with an increased airway responsiveness to a variety of stimuli. Having shown that ApoA‐IV serum levels decrease during allergic inflammation and that supplementation with ApoA‐IV prevents the influx of eosinophils into the airways, we further assessed the impact of ApoA‐IV on airway hyperresponsiveness. Therefore, HDM‐challenged/sensitized mice were treated daily i.p. with recombinant ApoA‐IV (10 µg/100 µL) or vehicle (A. dest.) from day 10 to day 14 (Figure 4D). On day 15, airway hyperresponsiveness to methacholine was recorded by spirometric measurements using a FlexiVent system. Strikingly, systemic application of ApoA‐IV to HDM‐treated mice diminished the methacholine‐induced increases in airway resistance by 70% (Figure 4E) and enhanced airway compliance up to 300% (Figure 4F) compared to the HDM control group. Thus, our results show unequivocally that systemic ApoA‐IV directly counteracts airway allergy in mice by inhibiting eosinophil recruitment and airway hyperresponsiveness.

### 3.8 ApoA‐IV is decreased in serum of allergic patients and accumulates in mucus during chronic rhinosinusitis

**Figure 5:**
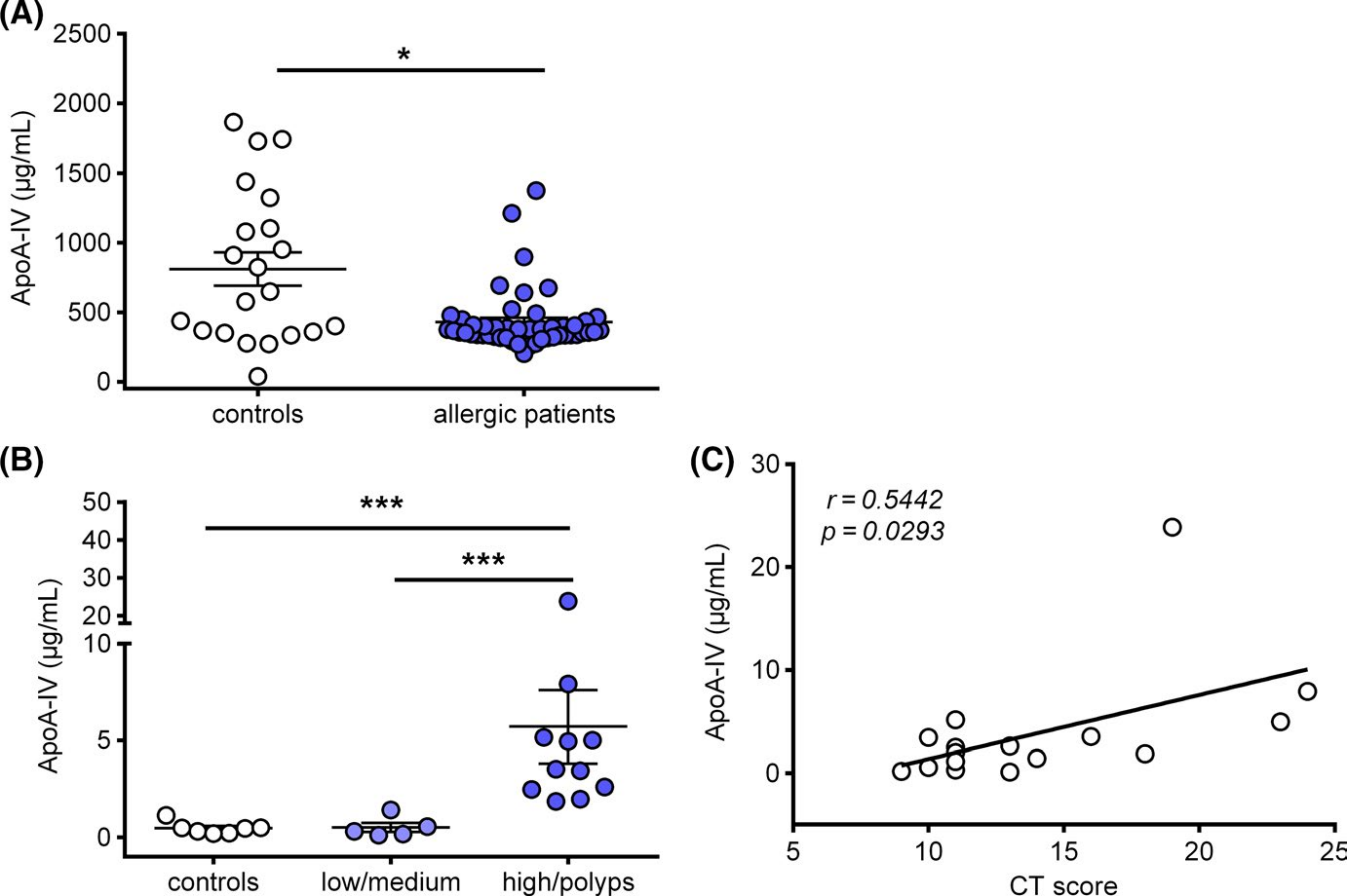
Apo‐IV serum levels are decreased in allergic patients and locally increased during CRS. (A) Serum ApoA‐IV concentrations were measured with ELISA in serum from 49 allergic patients and 17 healthy controls. (B) ApoA‐IV was detected by ELISA in mucus of 16 patients with rhinosinusitis and 7 healthy controls. (C) Pearson correlation of ApoA‐IV mucus levels and CT scores was statistically significant. Data are shown as individual values and in A and B as mean ± SEM; *P < .05,  ***P < .001, Student's *t* test or 1‐way ANOVA

ApoA‐IV levels have been shown to increase under immunotherapy in patients with allergic rhinitis.^28^ However, ApoA‐IV serum levels in allergic patients and healthy controls have not been compared yet. Hence, in this study we evaluated ApoA‐IV serum levels in 17 nonallergic healthy subjects and 49 untreated patients with respiratory allergic symptoms to aeroallergens (mainly grass pollen) (for further details please refer to Appendix S1, table E1). As presented in Figure 5A, ELISA analysis revealed consistently reduced ApoA‐IV concentrations in serum of allergic patients, with a mean value of 428.8 ± 31.02 µg/mL, whereas serum ApoA‐IV was 810.7 ± 120.1 µg/mL in healthy nonallergic controls. Albeit, no correlation between ApoA‐IV serum levels and laboratory parameters such as sIgE was found (data not shown).

Similar to allergy, chronic rhinosinusitis (CRS) is characterized by a pronounced eosinophilic inflammation of the lining of the nose and paranasal sinuses. Thus, we assessed ApoA‐IV levels in the mucus of CRS patients (for further details please refer to Appendix S1, table E2). Interestingly, ApoA‐IV levels correlated with their histology scores: Low mean ApoA‐IV levels of 0.47 ± 0.1 µg/mL and 0.55 ± 0.2 µg/mL were found in healthy controls and patients with low or medium clinical scores, respectively, whereas a mean mucus level of 5.28 ± 1.8 µg/mL was observed in patients with high histology scores or patients suffering from nasal polyps (CRSwNP) (Figure 5B). Moreover, a significant positive correlation was found between ApoA‐IV mucus levels and radiologic Lund‐Mackay^42^ scores (*r* = 0.5039; *P* = .033) (Figure 5C).

Thus, our data indicate that ApoA‐IV expression and/or metabolism is altered in allergic patients. Moreover, ApoA‐IV associates with the severity of inflammation in mucus of CRS patients.

## 4 DISCUSSION

In the present study, we demonstrate through several lines of evidence that apolipoprotein A‐IV bears potent anti‐allergic properties and thereby reveal a hitherto unknown anti‐inflammatory mechanism: First, we found that recombinant ApoA‐IV inhibits eosinophil responses to chemoattractants in assays of Ca^2+^ mobilization, shape change, integrin (CD11b) surface upregulation, and chemotaxis. The underlying molecular mechanism appears distinct from ApoA‐I and HDL‐induced signaling cascades as it occurs independently from ABCAI and SRBI binding, but is mediated through a novel pathway involving nuclear receptor NR1D1 (Rev‐ErbA‐α) and the protein kinases PI3K, PDK1, and PKA. Second, we established that ApoA‐IV specifically enhances apoptosis in eosinophils from allergic individuals but not healthy volunteers. Third, and in line with these in vitro data, systemic administration of ApoA‐IV prevented pulmonary eosinophilia and markedly improved airway hyperresponsiveness in a mouse model of HDM‐induced airway inflammation. And finally, we found that therapy‐naïve allergic patients have noticeably lower ApoA‐IV serum levels compared to healthy individuals. Moreover, we show that ApoA‐IV is present in mucus from CRS patients, where it might act in an anti‐inflammatory manner.

Up to now, low ApoA‐IV levels have been associated with serious conditions such as cardiovascular disorders^43^, ^44^, ^45^ and cancer.^46^, ^47^, ^48^ For instance, ApoA‐IV has been identified as a reliable biomarker in ovarian cancer.^49^, ^50^ Moreover, down‐regulated gene expression of ApoA‐IV has been demonstrated in patients with eosinophil‐driven ulcerative colitis^51^ and blunted ApoA‐IV responses to active lipid absorption after chronic high‐fat diet have been implicated in obesity and metabolic disorders.^52^, ^53^ However, the role of apolipoproteins in allergic conditions is still unclear. ApoA‐I, the main protein constituent of HDL, promotes cholesterol efflux from immune cells, such as neutrophils,^36^ monocytes,^54^ and macrophages,^55^, ^56^ and thereby attenuates their function. Recent findings from experimental mouse models suggest that ApoA‐I and ApoA‐I mimetic peptides might have therapeutic potential for allergic diseases.^57^, ^58^ Similarly, treatment with ApoE mimetic peptide alleviated eosinophilic airway inflammation and hyperresponsiveness in a mouse model of house dust mite‐induced asthma.^60^ Recent studies revealed that ApoA‐I is decreased in postanaphylactic patients,^61^ and serum levels of ApoA‐I were found to be positively correlated with FEV1 in subjects with allergic asthma.^62^ Conversely, a study in schoolchildren showed that high ApoA‐I is associated with the manifestation of asthma and atopy.^63^

Of interest, increased serum levels of ApoA‐IV were previously reported in patients with allergic rhinitis under allergen‐specific immunotherapy.^28^ We made the surprising observation that ApoA‐IV serum levels are noticeably reduced in therapy‐naïve allergic patients. However, the reasons for decreased ApoA‐IV levels under inflammatory and allergic conditions are a matter of speculation. Of note, Li et al reported that linoleic acid induces inflammatory cytokines such as TNF‐α and IL‐6, which in turn are able to reduce ApoA‐IV mRNA expression in hepatocytes^64^ and ApoA‐IV protein production in CaCo2 cells in vitro.^65^ Moreover, IL‐6 and TNF‐α are released in allergic responses and elevated levels of these cytokines have been demonstrated in bronchoalveolar fluid of asthmatic subjects.^66^, ^67^ However, the molecular mechanisms responsible for this association are still largely unexplored, but it is conceivable that there exists a link between chronic inflammation, ApoA‐IV levels and disease outcome in allergic patients.

Previous studies revealed an association of *APOA‐IV* gene variants with ApoA‐IV levels and increased risks for certain diseases such as coronary heart disease,^68^ renal diseases,^69^ depression,^70^ and obesity.^71^ For instance, Ser347 homozygotes have clearly lower ApoA‐IV plasma levels compared with carriers of the Thr347 allele and show a significantly increased risk of coronary heart diseases.^68^ Moreover, it was demonstrated that individuals who are homozygous for the Ser347 allele have higher BMI and percentage body fat compared with individuals homozygous for Thr347.^71^ Up to‐date no data are available whether *APOA‐IV* gene variants are also associated with a higher risk for chronic atopic diseases such as allergic asthma or rhinitis. In nonallergic patients with chronic rhinosinusitis, we provide evidence that anti‐inflammatory ApoA‐IV is not only present in nasal mucus but it is also correlated with the extent of inflammation. We assume that ApoA‐IV accumulates in the paranasal sinuses due to increased vascular permeability. However, it has been proposed that monocytes^72^ and dendritic cells^73^ are able to express ApoA‐IV, thus we cannot exclude that ApoA‐IV is also released locally by infiltrating inflammatory cells.

In our present work, we observed that ApoA‐IV potently affected effector cells of allergic inflammation such as eosinophils and neutrophils. Pretreatment of eosinophils with recombinant ApoA‐IV decreased their responses to chemoattractants by means of Ca^2+^ flux, shape change and integrin surface expression. Moreover, ApoA‐IV reduced eosinophil migration to baseline levels involving a signaling cascade mediated by Rev‐ErbA‐α, the NR1D1 (nuclear receptor subfamily 1, group D, member 1) gene product, which is a dominant transcriptional silencer that represses the expression of genes involved in numerous physiological functions, including circadian rhythm and metabolism,^74^ and plays a crucial role in maintaining immune functions.^75^, ^76^ For instance, inflammatory stimuli were shown to promote Rev‐ErbA‐α degradation in mice, and complete lack of Rev‐ErbA‐α further enhanced inflammation in the lungs following inflammatory challenge.^76^ In macrophages, Rev‐ErbA‐α decreased integrin expression and adhesion.^77^ In addition, it was recently shown that pharmacological activation of Rev‐ErbA‐α reduced lipopolysaccharide (LPS)‐induced neuro‐inflammation in mouse microglia in vitro and in vivo.^78^

In further experiments, we demonstrated that the ApoA‐IV‐induced signaling cascade involves the activity of PI3K, PDK1, and PKA. PI3Ks are a family of enzymes involved in cellular functions such as cell growth, proliferation, differentiation, motility, and migration.^79^ In numerous cell types, PI3K acts in a heterodimeric form consisting of one 85‐kDa regulatory and one 110‐kDa catalytic subunit. In previous work, we already elucidated the critical role for the PI3K/PDK1 cascade in transducing inhibitory signals on eosinophil effector function mediated by the prostaglandin E_2_ receptor EP4.^80^ In another study, ApoA‐IV has been identified to regulate food intake by acting as a satiation factor, which is released by, and is acting in, the hypothalamus.^39^ In this context, ApoA‐IV triggered the activation of the PI3K cascade in cultured primary hypothalamic neurons, and inhibition of PI3K signaling in rat brain noticeably decreased the potency of ApoA‐IV to reduce food intake.^39^ Moreover, cell culture experiments showed that ApoA‐IV improved glucose uptake in adipocytes by upregulating GLUT4 translocation in a PI3K‐dependent manner.^40^ These results further support our observation that ApoA‐IV engages with the PI3K signaling pathway to promote its anti‐inflammatory actions.

To confirm the in vivo relevance of the observed anti‐inflammatory activities of ApoA‐IV, we performed a well‐established mouse model of HDM‐induced airway inflammation. First, we revealed that HDM‐induced allergic inflammation in mice is accompanied by a significant drop in ApoA‐IV serum levels compared to healthy control mice. However, whether this effect is due to reduced ApoA‐IV synthesis from epithelial cells in the small intestine or due to increased ApoA‐IV degradation by the kidneys needs to be clarified in further studies. Of note, we could show that daily systemic treatment with ApoA‐IV for several days not only improved lung parameters, but also reduced eosinophil counts in the airways, spleen, and bone marrow of HDM‐challenged mice, suggesting that the ability of ApoA‐IV to inhibit airway inflammation is mediated by mechanisms that may include the attenuated expression of eosinophil survival factors such as IL‐5 and chemoattractants such as CCL11. Accordingly, Yao et al have shown that continuous application of the 5A ApoA‐I mimetic peptide inhibited the expression of IL‐4, ‐5, ‐10, ‐13 and ‐17 as well as the CC‐chemokines CCL7, ‐11, ‐17 and ‐24 in HDM‐challenged mice.^58^ This allows the conclusion that the therapeutic efficacy of apolipoproteins may be comparable with other anti‐eosinophilic drugs such as the monoclonal IL‐5 antibodies mepolizumab^81^ and reslizumab^82^ that provide significant and clinically relevant improvements in exacerbation rate and lead to a reduced use of oral corticosteroids in patients with severe eosinophilic asthma.

Moreover, previous work provided further evidence that ApoA‐IV is capable of inhibiting eosinophil‐driven inflammatory processes other than asthma. ApoA‐IV knockout mice exhibited a significantly greater inflammatory response in DSS‐induced colitis than did their wild type littermates. This greater susceptibility to DSS‐induced inflammation was reversed upon exogenous administration of ApoA‐IV. The authors proposed that ApoA‐IV is an endogenous anti‐inflammatory protein that acts via inhibition of P‐selectin‐mediated leukocyte and platelet adhesive interactions.^27^

In conclusion, our results unequivocally demonstrate the anti‐inflammatory properties of ApoA‐IV on effector cells of allergic inflammation. Further, we provide novel evidence that systemic elevation of ApoA‐IV protects against airway hyperresponsiveness, leukocyte infiltration into the airways and reduces eosinophil count in the circulation. Moreover, ApoA‐IV serum levels are significantly decreased in allergic patients and in HDM‐exposed mice. Thus, the present data collectively suggest that ApoA‐IV has promising diagnostic and therapeutic potential for allergic and inflammatory conditions, particularly those involving eosinophil effector functions.

## CONFLICTS OF INTEREST

AH received consultancy fees from AstraZeneca. All other authors declare no conflicts of interest.

## AUTHOR CONTRIBUTIONS

DR designed and performed experiments, analyzed data, interpreted the results and wrote the manuscript. AT, PL performed experiments and analyzed data. GJS, PVT and GM provided key material and interpreted the results. EMS and AH interpreted the results, supervised the study and edited the manuscript.
