# Supplementary material for: Apolipoprotein A‐IV acts as an endogenous anti‐inflammatory protein and is reduced in treatment‐naïve allergic patients and allergen‐challenged mice
Source: Allergy. 2019 Sep 10;75(2):392–402. doi: 10.1111/all.14022 (PMC7065107; doi:10.1111/all.14022)
Supplement: Supplementary file 8 [file ALL-75-392-s008.docx]

**Material**

All reagents were from Sigma (Vienna, Austria), unless speciﬁed. Assay buffer was made from Dulbecco`s modiﬁed PBS (with 0.9 mmol/L Ca^2+^ and 0.5 mmol/L Mg^2+^; Pan Biotech, Aidenbach, Germany), 0.1% BSA, 10 mmol/L HEPES, and 10 mmol/L glucose, pH 7.4. Human recombinant ApoA-IV was from Novoprotein (Summit, USA) and ApoA-I isolated from human plasma HDL. Human ApoA-IV ELISA was from Abcam (Cambridge, UK) and mouse ApoA-IV ELISA was from Cusabio (Houston, USA). Human eotaxin/CCL11 and IL-8 were from ImmunoTools (Friesoythe, Germany). FITC Annexin V Apoptosis Detection Kit I, anti-CD16-PE-Cy5, anti-CD193-PE and anti-CD11b-PE (ICRF44) were from Becton Dickinson (Vienna, Austria). Anti-CD14 BV421 and all anti-mouse antibodies were from BioLegend (San Diego, USA). The PKA inhibitor H-89 was from Cayman Chemical (Tallinn, Estonia), the PI3K inhibitor LY294002 was supplied by Biomol (Hamburg, Germany) and the PDK1 inhibitor BX-912 was from Axon Medchem (Groningen, The Netherlands. House dust mite extract was from Stallergenes Greer (Lenoir, USA). CellFix and FACS-Flow were from Becton Dickinson (Vienna, Austria). PVP-free polycarbonate filters were from Neuro Probe (Gaithersburg, USA). Fluo-3-AM was supplied from Life Sciences (Vienna, Austria).

Fixative solution was prepared by adding 9 ml of distilled water and 30 ml of FACS-Flow to 1 ml of CellFix.

**Patients and healthy controls**

Blood and serum were collected according to a protocol approved by the Ethics Committee of the Medical University of Graz (27-528 ex 14/15).

For functional assays, blood was collected on a daily basis from healthy and, for apoptosis assays, also from self-reported allergic volunteers.

Serum for ApoA-IV measurements was collected from 17 diagnosed healthy non-allergic and 49 diagnosed and untreated allergic volunteers. Allergic patients, who had been admitted to the outpatient clinic of the Department of Dermatology and Venereology of the Medical University of Graz because of allergic symptoms to aeroallergens, mainly grass pollen, were screened. Their personal history was taken and the current standard diagnostic procedures (intradermal tests, IgE determination by ImmunoCAP) were performed (Table 1). Specific and total IgE antibody levels in the patients’ sera were measured by using ImmunoCAP 250 (Thermo Fisher Scientific, Waltham, USA) according to the manufacturer’s instructions. Specific IgE values greater than 0.35 kU/L were considered positive.

Mucus was collected according to a protocol approved by the Ethics Committee of the Medical University of Graz (23-481 ex 10/11). Mucus was collected from 16 patients with chronic rhinosinusitis (CRS) and 7 healthy controls at the Department of Otorhinolaryngology of the Medical University of Graz. CRS was diagnosed according to the EPOS guidelines[^1^](#_ENREF_1). All CRS patients underwent a detailed history and physical examination, which included an allergy evaluation. All 16 patients had imaging computed tomography of their sinuses (Table 2). For grading CT scans the Lund‐Mackay classification was used, which relies on a score of 0–2 dependent upon the absence, partial or complete opacification of each sinus system and the ostiomeatal complex, deriving a maximum score of 12 per side[^2^](#_ENREF_2).

### Preparation of human peripheral blood leukocytes

Human leukocytes were isolated from blood samples of volunteers according to a protocol approved by the Institutional Review Board of the Medical University of Graz. In brief, erythrocytes were removed from citrated whole blood by dextran sedimentation for 30 min at RT. Polymorphonuclear leukocytes (pellet) were separated from mononuclear cells (buffy coat) by density gradient centrifugation (Histopaque 1077, Sigma–Aldrich) for 20 min at RT at 400 x g. Eosinophils were separated from neutrophils in the polymorphonuclear leukocyte fraction by negative magnetic selection using the MACS cell separation system (Eosinophil Isolation Kit, Miltenyi Biotech, Bergisch Gladbach, Germany). Therefore, non-eosinophils were indirectly magnetically labeled with a cocktail of biotin-conjugated monoclonal antibodies against CD2, CD14, CD16, CD19, CD56, CD123, and CD235a, as primary labeling reagent, and anti-biotin monoclonal antibodies conjugated to MicroBeads, as secondary labeling reagent. The magnetically labeled non-eosinophils were depleted by retaining them on a MACS® Column in the magnetic field of a Separator, while unlabeled eosinophils passed through the column with a resulting purity of typically ≥ 98 %.

**Mucus Collection**

An appropriate amount of mucus (minimum 0.2 mL) was harvested from each patient using a special mucus collection device (Sinus Secretion Collector, Medtronic-Xomed Inc., Jacksonville, FL) without doing any preoperative decongestion or irrigation. The mucus was collected under endoscopic guidance from the middle meatus and processed as described previously[^3^](#_ENREF_3). The mass of each mucus specimen was weighed, and a threefold excess of normal saline (0.15 M NaCl) was added. After vortexing, the mucus suspension was centrifuged, and the resulting supernatant fluid was frozen at -70°C.

### HDL isolation

HDL was isolated from plasma of healthy volunteers by density gradient ultracentrifugation as described[^4^](#_ENREF_4). Plasma density was adjusted with potassium bromide to 1.24 g/mL and a two-step density gradient was generated in centrifuge tubes (16 × 76 mm, Beckman) by layering the density-adjusted plasma (1.24 g/mL) underneath a NaCl-density solution (1.063 g/mL). Tubes were sealed and centrifuged at 65,000 rpm for 6 hours at 15^°^ C in a 90Ti fixed angle rotor (Beckman Instruments, Krefeld, Germany). After centrifugation, the HDL-containing band was collected and desalted via PD10 columns (GE Healthcare, Vienna, Austria) and immediately used for experiments.

**Shape change assay**

PMNL were pretreated with ApoA-IV or vehicle for 30 min and stimulated with serial dilutions of CCL11 for 20 min at 37 °C. Shape change was monitored by flow cytometry as the increase of forward scatter (FSC) and was expressed as percent of the vehicle response. Eosinophils distinguished from neutrophils due to their SSC properties and autofluorescence[^5^](#_ENREF_5).

**CD11b‐upregulation in eosinophils**

PMNL samples were pretreated with ApoA-IV or vehicle for 30 min and incubated with serial dilutions of CCL11for 30 min at 37°C. Samples were stained with anti‐CD16‐PE‐Cy5 and anti‐CD11b‐PE (ICRF44) Ab. Eosinophils were identified as CD16 negative cells. CD11b upregulation was analyzed by flow cytometry as previously described[^6^](#_ENREF_6).

**Calcium flux**

Isolated human eosinophils were loaded with the calcium sensitive dye Fluo‐3‐AM in the presence of 0.02% pluronic F‐127, pretreated with ApoA-IV or vehicle and stimulated with CCL11. Changes in intracellular Ca^2+^ were detected as fluorescence increase in the FL1‐(530/30 nm) channel[^7^](#_ENREF_7).

**Chemotaxis**

Purified eosinophils were used to study eosinophil chemotaxis, whereas neutrophil chemotaxis was performed separately with PMNL fractions. Cells were pretreated with ApoA-I, -IV, HDL or vehicle as indicated, placed into the top wells of a 48-well micro chemotaxis chamber and were allowed to migrate towards the respective chemoattractant for 1 hour at 37 °C[^8^](#_ENREF_8). PVP-free polycarbonate filter membranes with a pore size of 5 µm were used. Migrated eosinophils or neutrophils in the bottom wells were collected and then enumerated by flow cytometry as described previously[^7^](#_ENREF_7). Therefore, eosinophils and neutrophils were gated by their forward and side scatter properties and by autofluorescence.

In some experiments, eosinophils were treated additionally with specific blocking antibodies or protein kinase inhibitors for 30 min at 37 °C, cells were then exposed to ApoA-IV, ApoA-I, HDL or vehicle and chemotaxis was induced with CCL11. Concentrations and treatment conditions were chosen based on literature[^6^](#_ENREF_6)^,^ [^7^](#_ENREF_7)^,^ [^9^](#_ENREF_9).

### Cholesterol-rich microdomain (lipid raft) assessment

Isolated eosinophils were incubated with ApoA-IV, ApoA-I or vehicle for 60 min at 37°C; subsequently cells were washed with PBS and incubated with 1 μg/ml FITC-cholera toxin B for 60 min at room temperature. Eosinophils were fixed and lipid raft abundance was measured by flow cytometry[^10^](#_ENREF_10).

**CCR3 staining**

Isolated eosinophils were pretreated with ApoA-IV for 60 min at 37°C. Following stimulation cells were stained with PE-anti-CD193 (CCR3) antibodies or isotype control for 30 min at 4°C. Surface expression of CCR3 was evaluated by flow cytometry and expressed as % of CCR3 positive cells.

### Apoptosis assay

Apoptosis of eosinophils was determined as described previously[^11^](#_ENREF_11). Purified eosinophils were kept in RPMI (ThermoFisher Scientific) supplemented with IL-5 (50 pM), 1% FBS and PenStrep (Sigma-Aldrich) in the absence or presence of ApoA-IV (3 µg/mL). At various time points aliquots were removed and washed twice in PBS, and the cells were resuspended in binding buffer. Eosinophils were then stained with annexin V-FITC (1/100) and propidium iodide (PI) (1/50) in the dark for 10 min at room temperature according to the manufacturer’s protocol (Annexin V-FITC Apoptosis Detection Kit I, BD Pharmingen) and immediately analyzed by flow cytometry. Each sample was acquired for 1 min, and the total number of eosinophils gated on a forward scatter/side scatter plot and the percentage of non-apoptotic cells (annexin V^neg^/propidium iodide^neg^), early apoptotic cells (annexin V^pos^/propidium iodide^neg^), late apoptotic cells (annexin V^pos^/propidium iodide^pos^) and necrotic cells (annexin V^neg^/propidium iodide^pos^) was recorded.

**House dust mite (HDM)-induced allergic lung inflammation**

The HDM model was performed as previously described by Plantinga et al.[^12^](#_ENREF_12). Eight‐week‐old female Balb/c mice were used in this study. Mice were housed in individually ventilated cages (5 per cage) under controlled conditions of temperature (21 °C), air humidity (50%) and a 12 h light/dark cycle (lights on at 6:00 a.m.). Standard chow (altromin 1324 FORTI, Altromin, Lage, Germany) and water were provided *ad libitum*. The experimental procedure used in this study was approved by the Austrian Federal Ministry of Science, Research and Economy (protocol number: BMWFW-66.010/0020-WF/V/3b/2015) conform to Directive 2010/63/EU, and was performed in accordance with national and international guidelines. Mice were randomly assigned to the different treatments. Three cohorts of mice were investigated:

1. On day 1 the first group of mice was anesthetized with ketamine (100 mg/kg) and xylazine (10 mg/kg) via intraperitoneal injection and sensitized intranasally with 1 µg HDM protein in 40 µL PBS. Mice were then anesthetized with ketamine (100 mg/kg) and xylazine (10 mg/kg) via intraperitoneal injection and challenged by intranasal application of 10 µg HDM protein in 40 µL PBS from day 7 to day 11. On day 15, whole blood was collected by cardiac puncture and serum was prepared by centrifugation. ApoA-IV serum levels in untreated and HDM sensitized and challenged mice were determined by ELISA.

2. The second group of mice was sensitized and challenged as described above but received a daily i.p. injection of ApoA-IV (10 µg/100 µL) or vehicle (Aqua dest.) from day 7 to day 14. On day 15, mice were anesthetized with ketamine (100 mg/kg) and xylazine (10 mg/kg) via intraperitoneal injection, the trachea was exposed and a total 3 mL of BAL buffer (PBS containing 1 mM EDTA) divided into three parts was instilled into the lung and BAL fluid was collected and stored on ice. BAL fluid was centrifuged (400 x g, 4°C for 7 min) and collected cell pellets were subsequently subjected to a staining protocol. In brief, erythrocytes were lysed using an ammoniochloride solution. After a washing step, non-specific binding sites were masked using TruStain fcX™ antibody (anti-mouse CD16/32; BioLegend, San Diego, CA 1:100) for 15 min on ice, followed by incubation with specific anti-mouse antibodies: Siglec F (1:100), CD11b (1:200), CD11c (1:200) (all from BD Pharmingen), Ly6G (1:500) and MHC-II (1:200) (all from BioLegend) for 30 min at 4°C. Cells were washed, fixed and analyzed by a BD FACS Canto II flow cytometer. In detail, after doublet cells had been excluded, lymphocytes were identified as FSC/SSC^low^ CD11b^neg^/CD11c^neg^ cells. Remaining cells were further gated as CD11c^neg^/Siglec F^pos^ eosinophils and CD11c^pos^/Siglec F^pos^ alveolar macrophages (AM). CD11c/Siglec F double-negative cells were characterized as CD11b^pos^/Ly6G^pos^ neutrophils and Ly6G^neg^/Ly6C^pos^ monocytes.

3. The third group of mice was treated in an analogous manner as the second group, but received a daily i.p. injection of ApoA-IV (10 µg/100 µL) or vehicle (Aqua dest.) from day 10 to day 14. On day 15, these mice where anesthetized with ketamine (100 mg/kg) and xylazine (10 mg/kg) via intraperitoneal injection and airway hyperresponsiveness (AHR) to methacholine was recorded with the FlexiVent system (Scireq, Montreal, CA).

**Tables**

| **Variable** | **Controls (n=17)**  **mean or % (SD)** | **Patients (n=49)**  **mean or % (SD)** |
| --- | --- | --- |
| **Demographics** |  |  |
| Age, years | 23.9 (2.3) | 25.8 (11.6) |
| Gender (% females) | 53 % | 39 % |
| **Sensitization frequency (%)** |  |  |
| Grass | - | 100 |
| Birch | - | 51 |
| Ash | - | 12.2 |
| Ragweed | - | 8.2 |
| Mugwort | - | 18.4 |
| *Alternaria alternata* | - | 8.2 |
| House dust mite | - | 16.3 |
| Cat | - | 2 |
| Hazelnut | - | 2 |
| **IgE reactivity, mean kU/L (SD)** |  |  |
| Total IgE | 30.4 (36.8) | 197.9 (378.8) |
| Grass | - | 29.2 (34.7) |
| Birch | - | 20.9 (26.9) |
| Ash | - | 1.4 (0.5) |
| Ragweed | - | 7.3 (5.0) |
| Mugwort | - | 3.5 (7.2) |
| *Alternaria alternata* | - | 4.9 (5.3) |
| House dust mite | - | 4.1 (3.9) |
| Cat | - | 1.5 (0.0) |
| Hazelnut | - | 2.6 (0.0) |

**Table E1: Allergic patients and controls enrolled for ApoA-IV serum measurements.**

| **Variable** | **Controls (n=7)**  **mean or % (SD)** | | **Patients (n=16)**  **mean or % (SD)** |
| --- | --- | --- | --- |
| **Demographics** |  | |  |
| Age, years | 35 (7.6) | | 46 (10.7) |
| **Diagnosis (%)** |  | |  |
| CRSsP  CRSwP |  | | 31  69 |
| **Therapy (%)** |  | |  |
| Topical steroids (TS) |  | | 50 |
| Systemic steroids (SS)  TS+SS |  | | 6.25  18.75 |
| no |  | | 25 |
| **Histology (%)** |  | |  |
| Minimal sinusitis  Low grade  Medium grade  High grade  Polyps |  | | 6.25  25  6.25  6.25  56.25 |
| **Eosinophilia (%)**  No |  | | 18.75 |
| Low  Middle  High |  | | 12.5  12.5  56.25 |
| **Scores**  CT score (Lund- Mackay Score)  Total symptoms score (VAS) | |  | 14 (4.7)  4 (2.2) |

**Table E2: Rhinosinusitis patients and controls enrolled for ApoA-IV mucus measurements.**

**Figure E1. Defining the working dose of ApoA-IV, ApoA-I and HDL.** Purified eosinophils were pretreated with ApoA-IV (1-10 µg/mL) (*n*=12), ApoA-I (1-30 µg/mL) (*n*=11), HDL (10-100 µg/mL) (*n*=14) or vehicle for 30 min at 37 °C, placed into the top-wells of a 48-well microchemotaxis chamber and were allowed to migrate towards CCL11 (3 nM) for 60 min at 37 °C. Migrated cells were enumerated by flow cytometry. Chemotaxis is expressed as migrated eosinophils and shown as mean ± SEM.

**Figure E2. ApoA-IV potently inhibits eosinophil responsiveness.** (A) PMNL were pretreated with ApoA-IV (1 µg/mL) and stimulated with serial dilutions of CCL11 for 20 min. Shape change of eosinophils was recorded by flow cytometry. Eosinophils were distinguished from neutrophils by their autofluorescence as well as forward scatter and side scatter properties (*n*=5), (B) PMNL were pretreated with ApoA-IV (1 µg/mL) and stimulated with serial dilutions of CCL11 for 30 min. Samples were stained with anti‐CD16‐PE‐Cy5 and anti‐CD11b‐PE (ICRF44) Ab and CD11b upregulation was detected by flow cytometry (*n*=6). Eosinophils were identified as CD16-negative cells. (C, D) Purified eosinophils were pretreated with ApoA-IV (1 and 3 µg/mL) for 30 min and Ca^2+^ flux was induced by CCL11 (3 nM) and analyzed by flow cytometry. (*n*=6). (D) Data are calculated as stimulated – baseline difference. All data are shown as mean ± SEM; *** p*<0.0*5; two-way/one-way ANOVA.

**Figure E3. ApoA-IV does neither affect lipid raft abundance nor CCR3 expression in eosinophils.** (A) Lipid rafts were assessed in purified eosinophils using cholera toxin B-FITC and measured by flow cytometry (*n*=6-9). (B) CCR3 surface expression was determined in purified eosinophils by flow cytometry and expressed as % of CCR3 positive cells (*n*=9). Data are shown as mean ± SEM, (A) one-way ANOVA, (B) Student’s t-test.

**Figure E4. ApoA-IV differentially regulates apoptosis in eosinophils from allergic and non-allergic donors**. Purified eosinophils from allergic and non-allergic donors were resuspended in assay media, treated with ApoA-IV (3 µg/mL), ApoA-I (10 µg/mL) or vehicle and cultured for 18 h. After incubation, apoptosis was assessed by flow cytometry using annexin-V/PI staining. (A) Annexin-V-negative/PI-negative cells were considered live cells, (B) Annexin-V-positive cells were considered apoptotic cells, (C) Annexin-V-positive/PI-negative cells were considered early apoptotic cells, (D) Annexin-V-positive/PI-positive cells were considered late apoptotic cells and (E) Annexin-V-negative/PI-positive cells were considered necrotic cells. Data are expressed as % of total cells (*n*=5-7) and are shown as mean + SEM, **p<0.05*; one-way ANOVA.

**Figure E5. ApoA-IV serum levels are significantly reduced in HDM-sensitized and challenged mice.** On day 1, eight‐week‐old female Balb/c mice were sensitized intranasally with 1 µg HDM and challenged by intranasal application of 10 µg HDM per day from day 7 to 11. On day 15 whole blood was collected by cardiac puncture and serum was gained by centrifugation. ApoA-IV serum levels were determined by ELISA (*n*=5-7). Data are shown as mean ± SEM, ** p<0.05*; Student’s t-test.

**Figure E6. Treatment with ApoA-IV does not affect numbers of macrophages, lymphocytes, monocytes and neutrophils in BAL fluid of mice in HDM-induced airway inflammation.** On day 1, eight‐week‐old female Balb/c mice were sensitized intranasally with 1 µg HDM and challenged by intranasal application of 10 µg HDM per day from day 7 to 11. From day 7 to 14 mice were daily injected with 10 µg ApoA-IV or vehicle. On day 15 BAL fluid was taken from six mice per group and cellular contents were analyzed by flow cytometric staining. Data are shown as mean ± SEM; Student’s t-test.

**Figure E7. ApoA-IV protects mice from HDM-induced systemic eosinophilia.** On day 1, eight‐week‐old female Balb/c mice were sensitized intranasally with 1 µg HDM and challenged by intranasal application of 10 µg HDM per day from day 7 to 11. From day 7 to 14 mice were daily injected with 10 µg ApoA-IV or vehicle. On day 15 (A) splenocytes were prepared and (B) bone marrow cells were isolated from femur and tibia, and cell counts were analyzed by flow cytometric staining (*n*=4 per treatment group). Data are shown as mean ± SEM; ** p<0.05,* **** p<0.001*; Student’s t-test.

**References**

1. Fokkens WJ, Lund VJ, Mullol J, Bachert C, Alobid I, Baroody F, et al. European Position Paper on Rhinosinusitis and Nasal Polyps 2012. Rhinol Suppl 2012; 23:3 p preceding table of contents, 1-298.

2. Lund VJ, Mackay IS. Staging in rhinosinusitus. Rhinology 1993; 31:183-4.

3. Schmid C, Habermann W, Braun H, Gugatschka M, Oriel BS, Smietana JA, et al. Released intranasal eosinophilic major basic protein as a diagnostic marker for polypoid chronic rhinosinusitis. Otolaryngol Head Neck Surg 2010; 143:386-91.

4. Holzer M, Kern S, Trieb M, Trakaki A, Marsche G. HDL structure and function is profoundly affected when stored frozen in the absence of cryoprotectants. J Lipid Res 2017; 58:2220-8.

5. Schratl P, Sturm EM, Royer JF, Sturm GJ, Lippe IT, Peskar BA, et al. Hierarchy of eosinophil chemoattractants: role of p38 mitogen-activated protein kinase. Eur J Immunol 2006; 36:2401-9.

6. Konya V, Ullen A, Kampitsch N, Theiler A, Philipose S, Parzmair GP, et al. Endothelial E-type prostanoid 4 receptors promote barrier function and inhibit neutrophil trafficking. J Allergy Clin Immunol 2013; 131:532-40 e1-2.

7. Frei RB, Luschnig P, Parzmair GP, Peinhaupt M, Schranz S, Fauland A, et al. Cannabinoid receptor 2 augments eosinophil responsiveness and aggravates allergen-induced pulmonary inflammation in mice. Allergy 2016; 71:944-56.

8. Sturm EM, Schratl P, Schuligoi R, Konya V, Sturm GJ, Lippe IT, et al. Prostaglandin E2 inhibits eosinophil trafficking through E-prostanoid 2 receptors. J Immunol 2008; 181:7273-83.

9. Sturm EM, Parzmair GP, Radnai B, Frei RB, Sturm GJ, Hammer A, et al. Phosphoinositide-dependent protein kinase 1 (PDK1) mediates potent inhibitory effects on eosinophils. Eur J Immunol 2015; 45:1548-59.

10. Curcic S, Holzer M, Frei R, Pasterk L, Schicho R, Heinemann A, et al. Neutrophil effector responses are suppressed by secretory phospholipase A2 modified HDL. Biochim Biophys Acta 2015; 1851:184-93.

11. Hartnell A, Heinemann A, Conroy DM, Wait R, Sturm GJ, Caversaccio M, et al. Identification of selective basophil chemoattractants in human nasal polyps as insulin-like growth factor-1 and insulin-like growth factor-2. J Immunol 2004; 173:6448-57.

12. Plantinga M, Guilliams M, Vanheerswynghels M, Deswarte K, Branco-Madeira F, Toussaint W, et al. Conventional and monocyte-derived CD11b(+) dendritic cells initiate and maintain T helper 2 cell-mediated immunity to house dust mite allergen. Immunity 2013; 38:322-35.
